# Oxaloacetate enhances and accelerates regeneration in young mice by promoting proliferation and mineralization

**DOI:** 10.3389/fcell.2023.1117836

**Published:** 2023-02-24

**Authors:** Josue Jaramillo, Caroline Taylor, Rachel McCarley, Melissa Berger, Emily Busse, Mimi C. Sammarco

**Affiliations:** Department of Surgery, Tulane School of Medicine, New Orleans, LA, United States

**Keywords:** bone, oxaloacetate, limb regeneration, cell metabolism, digit regeneration

## Abstract

Cell metabolism coordinates the biochemical reactions that produce carbon and ATP in order for the cell to proliferate, differentiate, and respond to environmental changes. Cell type determines metabolic demand, so proliferating skeletal progenitors and differentiated osteoblasts exhibit different levels of cell metabolism. Limb regeneration is an energetically demanding process that involves multiple types of tissues and cell functions over time. Dysregulation of cell metabolism in aged mice results in impaired regeneration, a defect that can be rescued in part by the administration of oxaloacetate (OAA). A better understanding of how cell metabolism regulates regeneration in general, and how these changes can be modulated to benefit potential regenerative strategies in the future is needed. Here we sought to better understand the effects of OAA on young mice and determine whether the same mechanism could be tapped to improve regeneration without an aged-defect. We also asked which dosing time periods were most impactful for promoting regenerative outcomes, and whether these effects were sustained after dosing was stopped. Consistent with our findings in aged mice we found that OAA enhanced regeneration by accelerating bone growth, even beyond control measures, by increasing trabecular thickness, decreasing trabecular spacing, and improving the patterning by decreasing the taper, making the regenerated bone more like an unamputated digit. Our data suggests that the decrease in spacing, an improvement over aged mice, may be due to a decrease in hypoxia-driven vasculature. Our findings suggest that OAA, and similar metabolites, may be a strong tool to promote regenerative strategies and investigate the mechanisms that link cell metabolism and regeneration.

## Introduction

**FIGURE 1:**
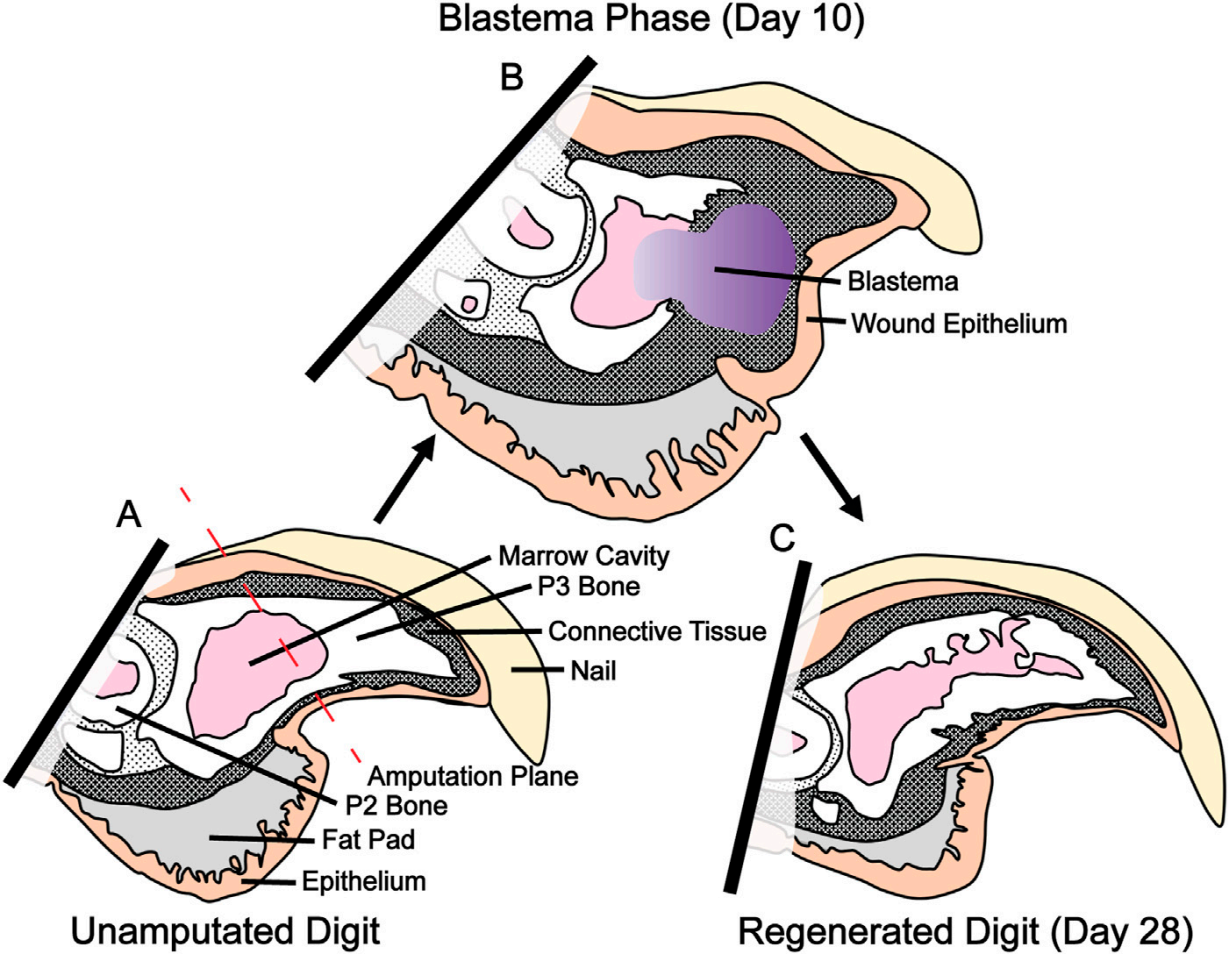
P3 amputation, blastema formation and regeneration. **(A)** Amputation through the third phalangeal element (P3) bisects the nail, epithelium, connective tissue, and P3 marrow cavity. **(B)** Blastema formation occurs between day 10 and 12. This heterogeneous fibroblast population will eventually become the regenerated bone and soft tissue. **(C)** Regeneration of bone and soft tissue is complete by day 28.

*De novo* limb regeneration is a highly desirable biological process resulting in the reformation of whole limb structures following injury. Capacity for limb regeneration varies widely between vertebrates. In mammals, regeneration is limited to the distal one-third of the third phalangeal element (P3). Amputation of the distal one-third of P3 results in a regenerative event which leads to reformation of both amputated bone and surrounding tissue (Figure 1). More proximal amputation fails to elicit a regenerative response and instead leads to the formation of a hypertrophic callus of bone (Bryant et al., 2002; Brockes and Kumar, 2005; Han et al., 2008; Fernando et al., 2011; Simkin et al., 2013). P3 regeneration occurs in a series of discrete phases, the first of which is degradation of residual bone followed by epidermal wound closure and blastema formation on days 7–10. Blastema formation results in a heterogenous mass of dedifferentiated cells which ultimately give rise to the regenerated bone and surrounding soft tissue. Bone regeneration begins on day 14 when blastema cells differentiate and form bone *via* direct intramembranous ossification (Han et al., 2008; Fernando et al., 2011; Simkin et al., 2013).

Limb regeneration is a metabolically demanding process in which proliferating progenitors and differentiated osteoblasts require extensive amounts of cellular resources and ATP. Energetic requirements of skeletal cells differ based on cell type, cellular environment, and progression along differentiation pathways (van Gastel and Carmeliet, 2021). Proliferating progenitors are heavily dependent upon glycolytic pathways and require high levels of both ATP for energy and carbon for biomass (Lunt and Vander Heiden, 2011; Salazar-Noratto et al., 2020). This glycolytic dependency is heightened in differentiated osteoblasts due to the metabolically demanding processes of matrix and amino acid synthesis (Bonewald, 2011; Capulli et al., 2014; Lee et al., 2020; van Gastel and Carmeliet, 2021). We showed that aging impairs the regeneration process, and that the regenerating digit shows high energetic demands with elevated levels of both glycolysis and oxidative phosphorylation. Oxaloacetate (OAA), a non-glycolytic intermediate, has been shown to enhance respiratory and glycolytic fluxes *in vitro* and *in vivo* in murine (Wilkins et al., 2014; Wilkins et al., 2016), as well as in human (Vidoni et al., 2021) neuronal cells, in addition to promoting regeneration in aged mice (Tower et al., 2022). Concomitantly enhancing respiratory and glycolytic fluxes should increase the cytosolic NAD^+^/NADH ratio, providing carbon to the mitochondria, and avoiding glycolysis feedback inhibition (Wilkins et al., 2016). OAA cannot be imported directly into the mitochondria and must use the malate-aspartate shuttle which first converts OAA to malate. Malate is imported across the mitochondrial membrane before being oxidized back to OAA again, generating NADH directly in the mitochondria for use in the electron transport chain (Lee et al., 2020). This shuttle has been shown to be necessary for active glycolysis in the osteoblast (Lee et al., 2020). Although only described thus far in neurons, these findings set the precedence for examining OAA-induced respiratory and glycolytic responses in other models and cell types.

*In vivo* administration of the anaplerotic metabolite OAA resulted in increases in both trabecular volume and thickness in aged mice and effected increases in WNT and TGFβ bone morphogenetic signaling pathways (Tower et al., 2022). In our previous study on aged mice, we administered OAA during both the blastema phase and the bone regeneration phase, and the measurable effects of OAA in aged mice were predominantly in trabecular thickness and bone volume (Tower et al., 2022). Here we build on our previous findings in aged mice to evaluate the effects of OAA in 8-week old young mice to establish whether the OAA-dependent regenerative outcomes we previously observed were age-dependent. We also investigated the timing of OAA dosing in both aged and young mice. We show that administration of OAA in young mice can not only enhance trabecular thickness and volume, but is also able to reduce trabecular spacing. This effect is dependent on dosing during both the blastema phase and the bone regeneration phase and appears to act by promoting cell-dependent effects in both the progenitor and osteoblast populations to enhance regeneration. These effects, in part, are sustained even after cessation of dosing. Together, these data suggest that the mechanisms underlying OAA-dependent enhanced regeneration are not, for the most part, age-dependent and instead are cell-type dependent. Ideally, these findings can be used to further refine timing and target specific cell lineage types.

## Materials and methods

### Ethics statement

All experiments were performed in accordance with the standard operating procedures approved by the Institutional Animal Care and Use Committee of Tulane University Health Sciences Center (Protocol #1483).

### Amputation and animal handling

Adult 8-week-old female CD1 mice were purchased from the Charles River Laboratory (Wilmington, MA, RRID: IMSR_CRL:22). Mice were anesthetized with 1%–5% isoflurane gas with continuous inhalation. The second and fourth digits of both hind limbs were amputated at the P3 distal level as previously described and regenerating digits were collected at days 14, 21, 28, and 42 for analysis (Sammarco et al., 2015; Busse et al., 2019; Hoffseth et al., 2021a; Hoffseth et al., 2021b; Tower et al., 2022). The third digit was used as an unamputated control. Oxaloacetate (OAA) (Sigma, 07753) was dissolved in saline (PBS) and pH-adjusted with NaOH to 7.0 to allow the OAA solution to drift toward alkaline levels as previously described (Tower et al., 2022), with the exception that mice were treated daily with 1 g/kg OAA in 200 µL *via* i.p. injection. The vehicle-treated group received saline. Mice were dosed from day 10 until day 28. Dichloroacetate (DCA) (Alfa, B21897) was dissolved in PBS. Mice were treated daily with 0.05 g/kg DCA (Liang et al., 2020) in 100 µL *via* i.p. injection. The vehicle treated group received PBS.

### Tissue collection and histology

Digits were harvested at specified time points and were fixed in zinc-buffered formalin (Z-fix, Anatech, Battle Creek, MI). Bone was decalcified for 48 h in formic acid based decalcifier (Decal I, Surgipath). Masson’s trichrome was performed as previously described and per the manufacturer’s directions (Tower et al., 2022).

### Cell culture and metabolic assays

P3 periosteal cells were harvested from the P3 bones of C57BL/6 mice at 8 weeks old. The mice were euthanized and the P3 bone was dissected at the joint away from P2 and surrounding connective tissue was removed. Bones were placed in a 96-well Agilent Seahorse plate (Agilent, Santa Clara, CA) and cultured for 1 week as previously described (Busse et al., 2019) and treated with 2 mM OAA for 24 h. Cells were evaluated using the Seahorse Mito Stress Test (Agilent, Santa Clara, CA) using the standard protocol after optimization of FCCP concentration. Final well concentrations for stressors were 1.0 μM oligomycin, 0.75 μM FCCP, and 0.5 μM rotenone/antimycin A (*N* = 4-5 wells per group). For the Seahorse Bioscience Glycolytic Rate Assay (Agilent, Santa Clara, CA), P3 cells were plated, cultured, and treated for 24 h with 2 mM OAA and evaluated using the Seahorse Bioscience Glycolytic Rate Assay Test (Agilent, Santa Clara, CA) using the standard protocol. Final well concentrations for stressors were 0.5 μM rotenone/antimycin and 50 mM 2-deoxy-D-glucose (*N* = 4-6 wells per group).

MC3T3-E1 cells (clone 4, ATCC, CRL-2593, RRID: CVCL_0409) were grown in media containing 10% FBS in ⍺-MEM (Gibco, #11885). Cells were seeded and differentiated in a 96-well Agilent Seahorse plate for 3 weeks using ⍺-MEM containing 10% FBS, 8 mM *β*-glycerophosphate, and 50 μg/mL ascorbic acid. Cells were treated with 2 mM OAA for 24 h prior to Seahorse analysis (Sigma, 07753). Cells were evaluated after 24 h using the Seahorse Bioscience Mito Stress Test using the standard protocol after optimization of cell seeding density and FCCP concentration. Final well concentrations for stressors were 1.0 μM oligomycin, 1 μM FCCP, and 0.5 μM rotenone/antimycin A (*N* = 23 wells per group). For the Seahorse Bioscience Glycolytic Rate Assay, MC3T3-E1 cells were seeded and differentiated in an Agilent Seahorse plate (Agilent, Santa Clara, CA) for 3 weeks using ⍺-MEM containing 10% FBS, 8 mM *β*-glycerophosphate, and 50 μg/mL ascorbic acid. Cells were treated with 2 mM OAA and evaluated 24 h after using the Seahorse Bioscience Glycolytic Rate Assay Test and Mito Stress Test (Agilent, Santa Clara, CA) using the standard protocol after optimization of cell seeding density and FCCP concentrations. Final well concentrations for stressors were 0.5 μM rotenone/antimycin and 50 mM 2-deoxy-D-glucose (*N* = 23 wells per group). For proliferation assays undifferentiated MC3T3 cells were seeded and incubated with 2 mM OAA and Click-iT EdU reagent for 48 h (Invitrogen, C10339) before fixing with 4% formaldehyde and stained according to the manufacturer’s protocol. For Osteoimage, cells were differentiated for 3 weeks with 2 mM OAA with media changes daily (Wilkins et al., 2014; Wilkins et al., 2016) before being assayed. The extent of mineralization was determined using an Osteoimage Mineralization Assay (Lonza, PA-1503) according to the manufacturer’s directions. Briefly, cells were washed with 1× PBS before being fixed with 4% (w/v) PFA for 20 min at RT. Samples were washed with Osteoimage wash buffer and incubated with 0.1 mL/well staining reagent in the dark for 30 min. After incubation, cells were washed with wash buffer and imaged using a Cytation5 with a 492/520 nm filter set. Total area of fluorescent signal was quantified for the entire well. *N* = 5 for each group. For Hypoxia Green cells were cultured with 2 mM OAA and differentiated for 3 weeks with media changes daily (Wilkins et al., 2014; Wilkins et al., 2016) before being assayed. Intracellular hypoxia was quantified using 5 µM Image IT Hypoxia Green (Thermo Fisher, I14833) according to the manufacturer’s directions and imaged as described for Osteoimage.

### Micro-computed tomography

*Ex vivo* μCT images of the digits were obtained using a Bruker SkyScan 1,172 scanner (Bruker, Kontich, Belgium) at a pixel size of 4 µm with 0.2 rotation angle and five frame averaging using a custom 0.25 mm aluminum filter. The X-ray source used was 50 kV, 201 μA, and 10 W, as described previously (Tower et al., 2022). All samples were reconstructed using NRecon with smoothing correction disabled, a beam hardening correction of 24%, and a dynamic range of 0.00–0.339. Reconstructed digits were exported as 8-bit BMP output files, rotated transaxially in DataViewer, and binarized and 3D analyzed in CTAn. Global thresholds were used for all young mice (8-week old) data sets with minimum threshold value set to 0 and maximum threshold value of 67 and a global threshold for aged mice (18-month old) data sets with minimum threshold value set to 0 and maximum threshold value of 79. For analysis of hypomineralized tissue at day 14 we used CTAn (Bruker, Kontich, Belgium, RRID:SCR_021338) and the Binary Selection preview window to identify newly regenerated bone. Hypomineralized tissue was isolated by identifying mineralized areas below 67 (the 6-month old mouse threshold for bone in the digit) in the grayscale data stack. To exclude mineralized bone the Binary Selection preview window was used to set a global threshold with a lower bound of 35 and an upper bound of 67. The Morphological Operations plugin was used to remove the partial volume effect in the 3D binarized image using Opening, Round Kernel, and Radius of 1. The volume of hypomineralized tissue was quantified using 3D analysis. The taper of the digit morphology was quantified as previously described (Tower et al., 2022). Briefly, we identified the start of newly regenerated bone using CTAn and measuring the area of the newly regenerated bone from the P3 cortical bone stump to the distal tip. The bone area was recorded and graphically represented along the length of the newly regenerated bone for control and OAA treated samples (Tower et al., 2022). Bone mineral density (BMD) was calculated as previously described (Hoffseth et al., 2021a). Briefly, we calibrated attenuated X-ray data values from digit data sets to known mineral density standards of 0.25 and 0.75 mg calcium hydroxyapatite (CaHA) known as “phantoms” to determine the density of CaHA g/cm^-3^ in mineralized tissue (Hoffseth et al., 2021a).

### Statistical analysis

Statistical analysis was performed using GraphPad Prism (version 8, RRID:SCR_002798) (GraphPad, San Diego, CA). Micro-CT data: Eight digits were precluded from the analysis due to breakage, as determined by visual inspection of 3D digit renderings. Unless otherwise stated outliers were defined as having a Studentized residual >3.0 or < −3.0, or an unstandardized residual greater or less than three times the group interquartile range and removed. Normality of each group was tested using the Shapiro-Wilk test. Homogeneity of variances was tested using Levene’s test of equality of variances. Bone morphometrics were determined by unpaired Student’s t-test or two-way ANOVA for multiple timepoints. Following the omnibus two-way ANOVA, individual differences between age groups at each time point were determined by post-hoc comparisons using Bonferroni’s adjustment for multiple comparisons (Tower et al., 2022). For all tests, *p* ≤ 0.05 was considered significant. The data are presented as the means ± SD. Seahorse data: The statistical significance of the differences in the means of the investigated cell lines was determined by unpaired Student’s t-test (Tower et al., 2022). For all tests, *p* ≤ 0.05 was considered significant. The data are presented as the means ± SEM.

## Results

### OAA enhances skeletal regeneration in young mice

**FIGURE 2:**
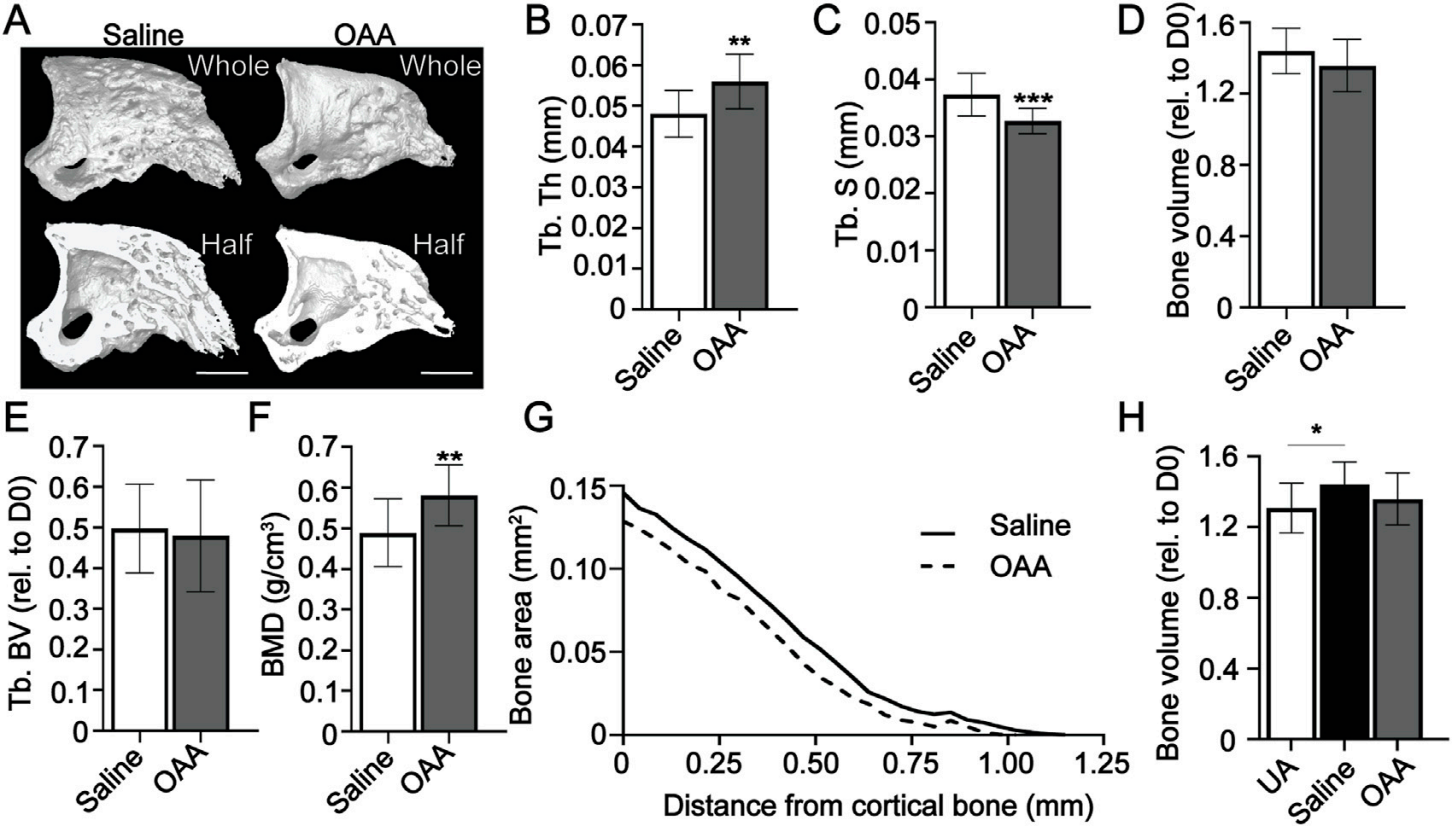
OAA enhances skeletal regeneration in young mice. **(A)** Radiographic images of whole and bisected digits in young mice at day 28 following administration of saline (Control) and OAA from day 10–28 following amputation of the distal P3. Bar 200 µm. Micro-CT quantification of **(B)** trabecular thickness (Tb. Th), **(C)** trabecular spacing (Tb. Sp), **(D)** bone volume (relative to day 0), **(E)** trabecular bone volume (Tb. BV) (relative to D0), **(F)** and bone mineral density (BMD). **(G)** 2D micro-CT analysis of regenerated bone from the remaining P3 cortical bone stump extending distally from saline control and OAA-treated mice. **(H)** Bone volume (relative to day 0) of saline controls and OAA-treated mice at day 28 compared to the bone volume of unamputated digits (UA). *n* = 15 digits/group. Graphs represent average values ± SD. *p < 0.05, **p < 0.01, ***p < 0.001.

We have previously shown that administration of OAA in aged mice increases both bone volume and trabecular thickness (Tower et al., 2022). To build upon this, we hypothesized that OAA-dependent metabolic pathways were not unique to aged mice and could potentially be exploited in young mice to likewise improve regeneration. We administered OAA daily to young 8-week old mice starting at the blastema phase (D10) through regeneration (D28). Morphometric analysis and 3D micro-CT image analysis (Figure 2A) at day 28 showed significant increases in trabecular thickness (Figure 2B) and decreases in trabecular spacing (Figure 2C) with no change in either total volume or trabecular volume (Figures 2D, E). Bone mineral density measurements at day 28 confirmed that administration of OAA significantly increased bone mineral density (BMD) compared to controls (Figure 2F). 2D analysis of the circumference of the regenerated bone showed that the regenerated bone in the OAA treatment group tapers more distally than in the saline treated group (Figure 2G), indicating that the bone narrows distally supporting that bone is built distally and not laterally (Tower et al., 2022). The volume of the regenerated digit typically overshoots the original unamputated bone volume. Unlike the control group, the bone volume at day 28 of the OAA treatment group does not overshoot the original unamputated volume (Figure 2H). These data indicate that both the thickness and spacing parameters are improved in OAA-treated young mice and that the regenerated bone structure of OAA-treated young mice more closely resembles that of the original unamputated digit with bone volume and taper that is more akin to the original patterning.

### OAA-enhanced regeneration is dependent upon dosing during both the blastema phase and the bone regeneration phase

**FIGURE 3:**
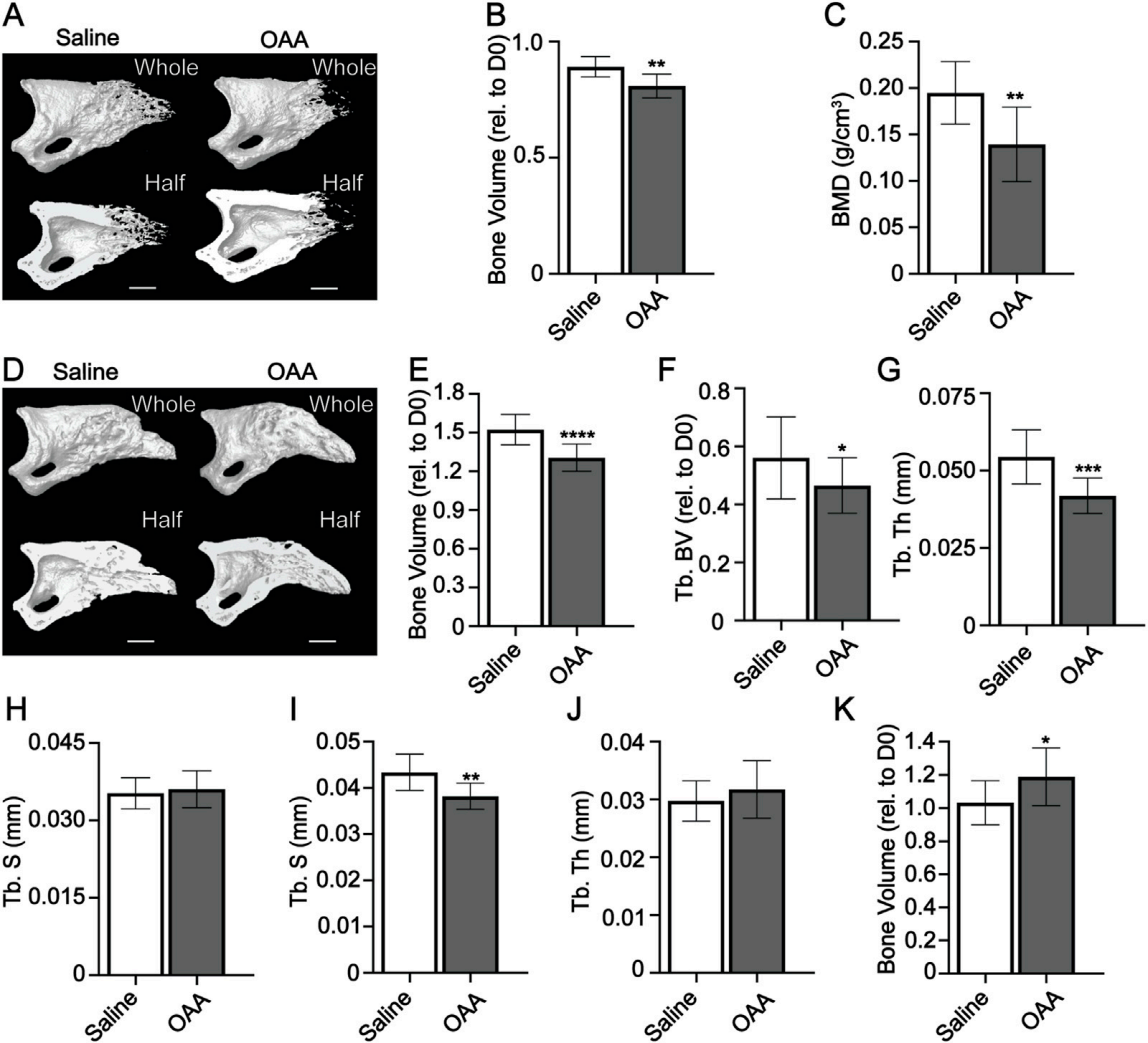
OAA-enhanced regeneration is dependent upon dosing during blastema and bone phases. **(A)** Radiographic images of whole and bisected digits in young mice at day 14 following administration of saline (Control) and OAA from day 10–14 following amputation of the distal P3. Bar, 200 µm. **(B)** Bone volume (relative to day 0). **(C)** Bone mineral density (BMD) at day 14 in mice treated with saline and OAA from day 10–14. **(D)** Radiographic images of whole and bisected digits in young mice at day 28 following administration of saline (Control) and OAA from day 14–28 following amputation of the distal P3. Bar, 200 µm. Micro-CT quantification of **(E)** bone volume (relative to day 0), **(F)** trabecular bone volume (Tb. BV) (relative to D0), **(G)** trabecular thickness (Tb. Th), and **(H)** trabecular spacing (Tb. Sp) at day 28 in mice treated with saline (Control) and OAA from day 14–28. Micro-CT quantification of **(I)** trabecular spacing (Tb. Sp), **(J)** trabecular thickness (Tb. Th), and **(K)** bone volume (relative to day 0) at day 21 in mice treated with saline (Control) and OAA from day 10–21. *n* = 10–16. Graphs represent average values ± SD. *p < 0.05, **p < 0.01, ***p < 0.001, ****p < 0.0001.

We previously showed that OAA is not able to accelerate bone regeneration in aged mice (Tower et al., 2022). To explore the influence of OAA administration on accelerating bone regeneration in young mice we dosed with OAA daily from blastema formation (day 10) to early bone formation (day 14) and found a significant decrease in bone volume and bone mineral density at day 14 (Figures 3A–C). We also evaluated daily dosing of OAA during the bone formation stage (day 14–28) without dosing during the blastema phase. Dosing with OAA during the bone formation phase reduced bone volume and trabecular volume and thickness but did not significantly affect trabecular spacing (Figures 3D–H). Evaluation of OAA dosing during the bone formation phase in 18-month aged mice (day 21–28) showed no change in any parameter (Supplementary Figure S1) (Tower et al., 2022). Next we wanted to see at what stage enhanced bone regeneration is detectable in young mice, so we dosed from day 10 to day 21 with OAA and evaluated bone parameters at day 21. We found that while trabecular spacing was significantly reduced in OAA-treated mice, trabecular thickness and whole bone volume showed no significant difference when compared to control (Figures 3I–K). Together these data suggest that, like aged mice, young mice require OAA administration during both the blastema phase and the bone regeneration phase to accelerate regeneration. While significant bone regeneration differences are first detected at day 28 in OAA-treated mice, the majority of bone gain is seen between day 14 and 21 (Supplementary Figure S2) (Tower et al., 2022). We next asked whether the effects of OAA were sustained after cessation of OAA dosing. We administered OAA from day 10–28 and then stopped dosing and analyzed digits on day 42. Evaluation of morphometric digit parameters at day 42 showed that there was no significant difference between the control and treated mice (Supplementary Figure S3).

### OAA promotes proliferation and delays mineralization

**FIGURE 4:**
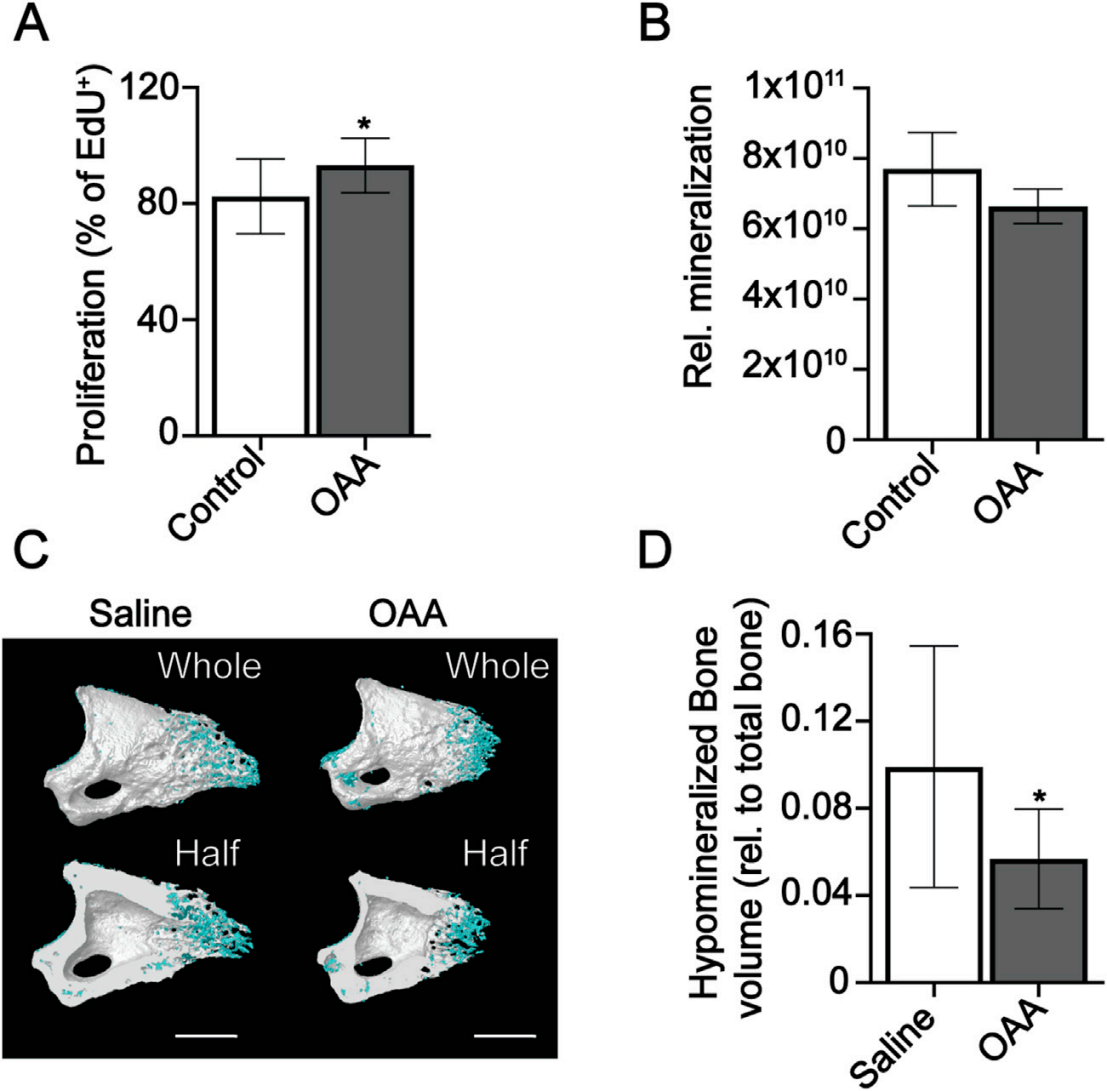
OAA increases proliferation and delays mineralization. **(A)** Proliferation of MC3T3 undifferentiated osteoprogenitors in OAA-treated and untreated cells. Graph represents mean number of EdU^+^ cells/total cells±SD. *n* = 11-12. *p < 0.05. **(B)** Quantification of mineralization in OAA-treated and untreated differentiated MC3T3 cells. Graph represents mean Osteoimage pixel intensity/area. *n* = 4-5. **(C)** Radiographic images of whole and bisected digits in young mice at day 14 following administration of saline (Control) and OAA from day 10–14 following amputation of the distal P3. Mineralized bone shown in white, hypomineralized bone shown in blue. Bar 200 µm. **(D)** Quantification of hypomineralized bone (relative to total bone). *n* = 8–12. *p < 0.05.

Given the differential effects of OAA on the heterogeneous blastema, and bone regeneration phases, in young and aged mice (Tower et al., 2022) we wanted to more closely examine the effects of OAA specifically on osteoprogenitors and differentiated osteoblasts. To investigate proliferation and mineralization *in vitro*, we used MC3T3 osteoprogenitor cells in the presence and absence of OAA. Treatment with OAA increased proliferation in osteoprogenitor cells (Figure 4A). Quantification of mineralization using OsteoImage showed treatment with OAA decreased pixel intensity (Figure 4B). We hypothesized that OAA may be increasing the amount of hypomineralized matrix (Dillon et al., 2021) produced *in vivo*, resulting in less mineralized bone at earlier stages, but more hypomineralized matrix for mineralization at later regenerative stages. To determine whether this was the case, we reduced our grayscale threshold and evaluated hypomineralized tissue at day 14 to determine whether there might be enhanced osteoid bone formation not detected as bone volume. However, our analysis showed that OAA-treated mice demonstrated both reduced hypomineralized tissue (Figures 4C, D) and reduced bone volume (Figure 3B) at day 14.

### OAA differentially modulates cellular metabolism and hypoxia in osteoprogenitors and differentiated osteoblasts

**FIGURE 5:**
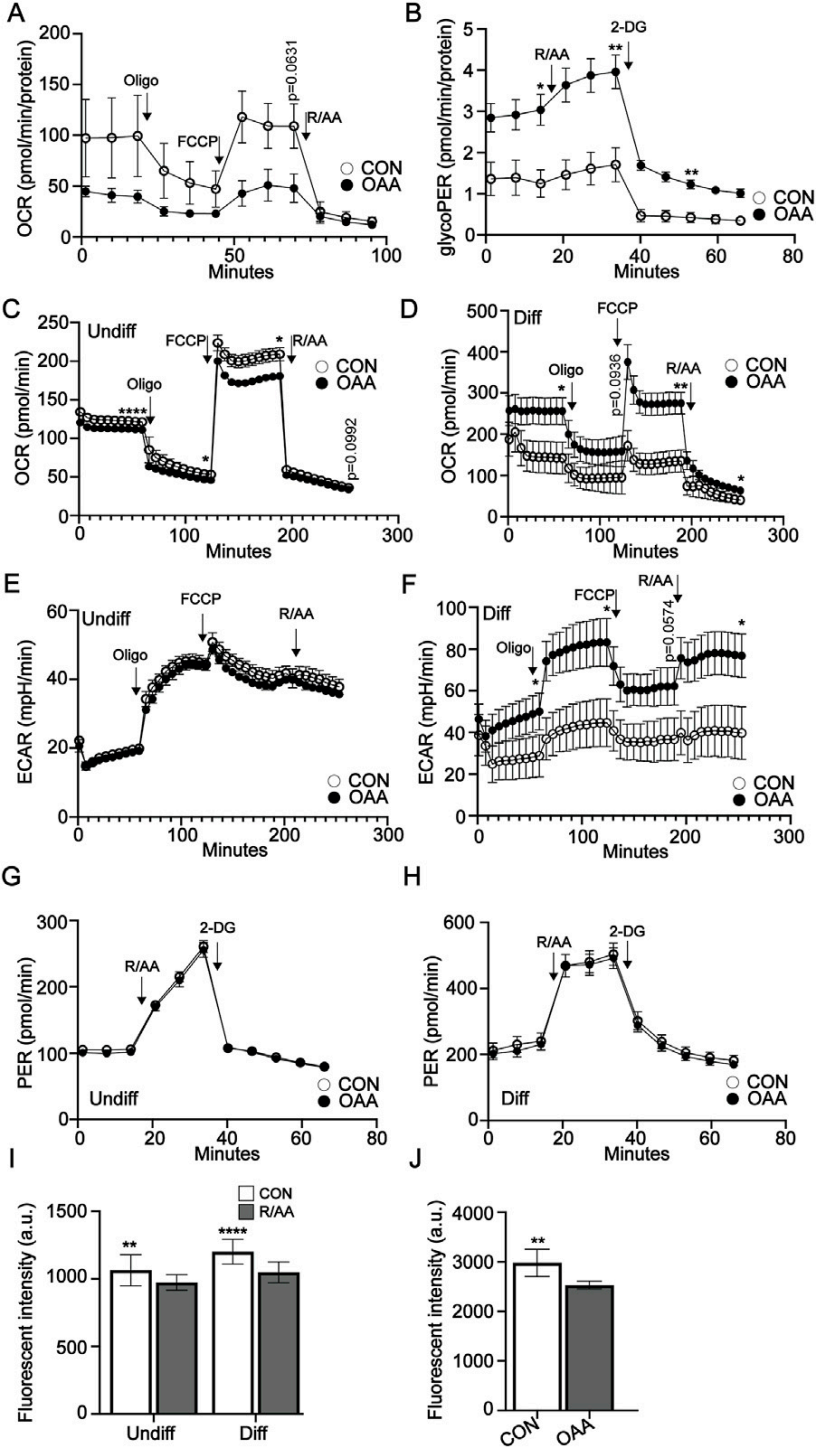
OAA-dependent modulation of cell metabolism and hypoxia. **(A)** Oxygen consumption rate (OCR) in P3 periosteal cells with and without OAA treatment. *n* = 4-5. **(B)** Glycolytic rate assay (GlycoPER) of P3 periosteal cells with and without OAA treatment. *n* = 4–6. Graphs represent average values ± SEM. OCR in **(C)** undifferentiated osteoprogenitors and **(D)** differentiated MC3T3 cells after treatment with OAA. Extracellular acidification rate (ECAR) in **(E)** undifferentiated osteoprogenitors and **(F)** differentiated MC3T3 cells after treatment with OAA. GlycoPER of **(G)** undifferentiated and **(H)** differentiated MC3T3 cells with or without OAA treatment. *n* = 9–24. Graphs represent average values ± SEM. **(I)** Quantification of intracellular hypoxia in osteoprogenitor and differentiated MC3T3 cells following inhibition of the electron transport chain using rotenone/antimycin A (R/AA). Graphs represent average values ± SD. *n* = 5 samples/condition. **(J)** Quantification of intracellular hypoxia in osteoprogenitor MC3T3 cells after treatment with OAA. Graphs represent average values ± SD. *n* = 5 samples/condition. *p < 0.05, **p < 0.01, ****p < 0.0001. *p* < 0.1 indicated on graph.

Cell metabolism is known to be differentially regulated in osteoprogenitors and osteoblasts to address specific cell functions (Guntur et al., 2014; Lee et al., 2017; Shen et al., 2022). To determine the impact of OAA on cell metabolism in endogenous osteoprogenitors we conducted Seahorse analysis on P3 periosteal cells, given that the periosteum is known to contribute the majority of osteoprogenitor cells during P3 regeneration (Dawson et al., 2018). We used Seahorse analysis to investigate oxidative phosphorylation (OxPhos) and glycolysis. Treatment with OAA showed a trend toward decreased maximal respiration in P3 periosteal cells (Figure 5A). Analysis using the Glycolytic Rate Assay showed increased basal glycolysis and compensatory glycolysis in response to treatment with OAA (Figure 5B). Given that cell metabolism is known to affect both osteoprogenitors and differentiated osteoblasts, we again used MC3T3 osteoprogenitor cells, differentiated them in the presence and absence of OAA, and used Seahorse analysis to investigate OxPhos and glycolysis. Treatment with OAA decreased maximal respiration in osteoprogenitor cells (Figure 5C) and increased maximal respiration in differentiated osteoblasts (Figure 5D). Extracellular acidification rate (ECAR) from the Mito Stress Test (Figures 5E, F) and analysis using the Glycolytic Rate Assay showed no change in glycolysis (Figures 5G, H), suggesting that OAA predominantly impacts OxPhos and not glycolysis. Previous studies suggest increases in cell metabolism are linked to increases in intracellular hypoxia (Prior et al., 2014; Kurokawa et al., 2015). To test whether OAA-driven changes in cell metabolism could potentially modulate changes in intracellular hypoxia we treated osteoprogenitors and differentiated osteoblasts with Rotenone/Antimycin A, inhibitors of mitochondrial complexes I and III required for O_2_ consumption *via* OxPhos and evaluated intracellular hypoxia using Hypoxia Green staining (Figure 5I). These results confirm that oxygen consumption *via* oxidative ATP synthesis contributes to intracellular hypoxia, resulting in significantly reduced Hypoxia Green staining following treatment with inhibitors. Treatment of MC3T3 preosteoblasts with OAA also resulted in decreased staining of Hypoxia Green (Figure 5J), paralleling our findings of OAA-dependent decreases in OCR (Figure 5C). Together these findings support that, as with aged mice (Tower et al., 2022), OAA differentially affects undifferentiated and differentiated osteoblasts, and demonstrate that OAA-dependent changes in OxPhos may be contributing to changes in intracellular hypoxia in osteoprogenitors.

### Dichloroacetate has no significant impact on digit regeneration

**FIGURE 6:**
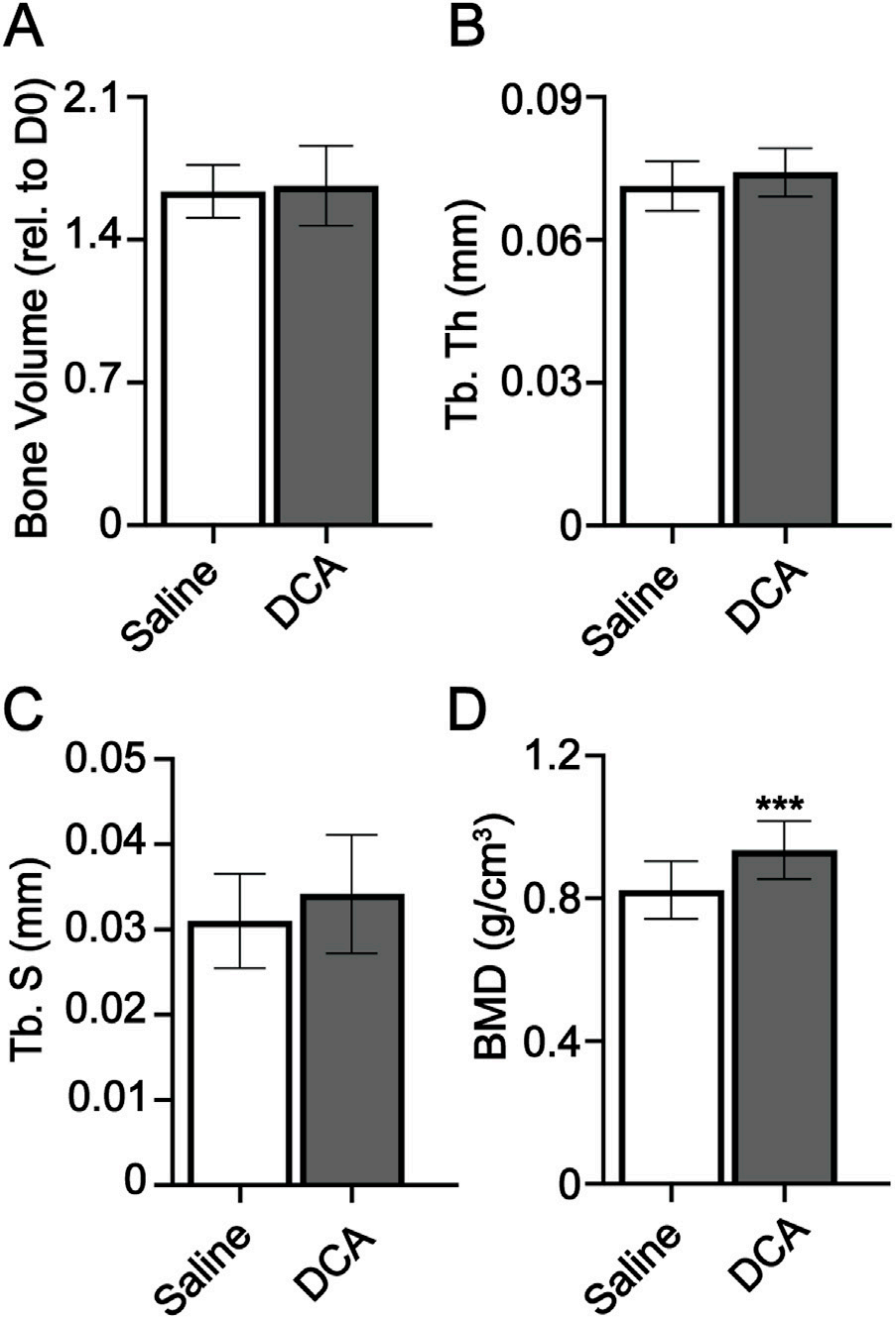
Dichloroacetate has no significant impact on digit regeneration. Micro-CT quantification of **(A)** bone volume (relative to day 0), **(B)** trabecular thickness (Tb. Th), **(C)** trabecular spacing (Tb. Sp), and **(D)** bone mineral density (BMD). *n* = 16–18 digits/group. Graphs represent average values ± SD. ***p < 0.001.

To determine whether the regenerative process was responsive to other approaches to manipulating cell metabolism, we investigated the impact of dichloroacetate (DCA) on regeneration. DCA is a mitochondria-targeting small molecule (Bonnet et al., 2007; Pan and Mak, 2007) that inhibits pyruvate dehydrogenase kinase, increasing the flux of pyruvate into the mitochondria and promoting OxPhos over glycolysis. DCA has been used in several models with systemic effects in multiple tissues as a means of rerouting cell metabolism (Michelakis et al., 2008). Mice were administered DCA daily from day 0 to day 28. Micro-CT analysis at day 28 showed no significant difference in bone volume, trabecular thickness, and trabecular spacing, but showed an increase in BMD (Figures 6A–D). Our data support that regeneration, while impacted by manipulation of cell metabolism, is not impacted by manipulation of all metabolic pathways.

## Discussion

This study sought to investigate whether the OAA-dependent enhancement of bone regeneration in aged mice (Tower et al., 2022) is conserved in young mice. In young mice, the OAA-treated group showed enhanced regeneration with increased distal taper in the regenerated bone and no significant difference in bone volume when compared to the control group. This suggests that OAA-treated digits were more like the original unamputated digit than regenerated digits in the control group. Additionally, unlike aged mice (Tower et al., 2022), trabecular spacing was decreased in OAA-treated digits. Similar to aged mice (Tower et al., 2022), these phenotypic changes required administration of OAA during both the blastema and the bone formation stages. Independent dosing in the blastema and bone formation phase did not promote regeneration. In fact, administration of OAA *in vivo* during the early progenitor blastema phase (day 10–14) decreased bone regeneration compared to the control group. These findings were supported by our *in vitro* studies showing that OAA supports proliferation in favor of differentiation and mineralization *in vitro*. This OAA-dependent lag in bone formation *in vivo* is followed by accelerated bone formation in the late stages of regeneration between days 21 and 28, as with the aged mice (Tower et al., 2022).

While the OAA-dependent impact on regeneration in young mice parallels our findings in aged mice (Tower et al., 2022), young mice also demonstrated decreased trabecular spacing. In aged mice we hypothesized that the inability to resolve OxPhos-driven hypoxia and downstream VEGF signaling resulted in increased angiogenesis (Tower et al., 2022) and therefore increased trabecular spacing. We hypothesized this may also be true in young mice. Our *in vitro* data support that OAA differentially affects cell metabolism in osteoprogenitors and differentiated MC3T3 cells, decreasing OxPhos in osteoprogenitors and increasing OxPhos in differentiated osteoblasts. This trend toward OAA-dependent decreased respiration was also evident in P3 periosteal cells. Lending support to this hypothesis is the fact that reduced OxPhos in progenitor osteoblasts effected a decrease in intracellular hypoxia in osteoprogenitor cells. Conversely, differentiated MC3T3 cells showed an increase in OxPhos with OAA, with no significant change in intracellular hypoxia. While we cannot conclusively say that differences in the spacing phenotype in aged and young are driven by intracellular hypoxia, our findings suggest that further research should be done to investigate this mechanistic link. Proliferating progenitors require ATP for energy and carbon for biomass (Lunt and Vander Heiden, 2011) and are highly glycolytic (Salazar-Noratto et al., 2020). This glycolytic dependency increases in differentiated osteoblasts due to the high metabolic demand of matrix synthesis and amino acids (Bonewald, 2011; Capulli et al., 2014; Lee et al., 2020; van Gastel and Carmeliet, 2021). The reciprocal metabolic data evaluating glycolysis showed that while OAA did not significantly change the Proton Efflux Rate in either MC3T3 progenitor or MC3T3 differentiated cells, our studies in P3 periosteal cells show a significant OAA-dependent increase in both basal glycolysis and compensatory glycolysis. Taken together, our cell metabolism data support that OAA has a differential impact on both progenitor and differentiated osteoblasts, and that this may be independently beneficial to promote proliferation during a progenitor stage and mineralization during differentiation.

Our current study in young mice also offers insight into the dosing schedule and impact window of OAA. Bone morphometric parameters in the control group of mice were equivalent to the OAA-treated group at day 42 when dosing was stopped and digits were allowed to continue remodeling. We cannot completely rule out the fact that continued dosing from day 10–42 in young mice would have resulted in a significant difference in bone morphometric parameters between the control group and the OAA-treated group. However, the fact that the majority of bone growth occurred between days 10 and 21 in early bone formation stages (similar to aged mice) (Supplementary Figure S2) and that dosing from day 10–42 in aged mice did not show a significant difference in morphometric parameters between the control group and OAA-treated group (Supplementary Figure S4) (Tower et al., 2022) suggest that the window of influence for OAA occurred between day 14 and 28 and that the control mice “caught up” to the accelerated bone regeneration in the OAA-treated group, and that the regenerative impact of OAA did not reverse after dosing. While OAA-enhanced regeneration did not result in a significant difference in bone parameters at day 42, accelerated bone growth is a highly desirable effect in treatments, particularly when the bone regrowth is finite and not unrestricted. Further, in a spatially and temporally dynamic model such as limb regeneration, tandem targeted treatments for each phase of regeneration stand to be far more advantageous than a single treatment alone. A regenerative accelerant could potentially be used in combination with a second late-acting bone stimulant. OAA was specific in enhancing regeneration, given that DCA was unable to effect significant changes in the bone morphometric parameters. DCA was, however, able to increase BMD. This suggests that while OAA may not be effective alone in building bone beyond endogenous regeneration, it holds potential as an accelerant for bone regeneration and could be used in conjunction with other compounds. Similarly, while DCA is unable to modulate morphometric parameters it could be used to increase BMD in conjunction with other compounds that do influence bone architecture. While our data show that OAA can indeed be impactful in bone regeneration in young mice, the exact mechanism is yet to be determined.

## Data Availability

The raw data supporting the conclusions of this article will be made available by the authors, without undue reservation.

## Abbreviations

BMD, Bone Mineral Density; DCA, Dichloroacetate; ECAR, Extracellular Acidification Rate; GlycoPER, Glycolytic Proton Efflux Rate; P3, third phalangeal element; Micro-CT, Micro-computed Tomography; OAA, Oxaloacetate; OCR, Oxygen Consumption Rate; OxPhos, Oxidative Phosphorylation.
