# Supplementary material for: Oxaloacetate enhances and accelerates regeneration in young mice by promoting proliferation and mineralization
Source: Front Cell Dev Biol. 2023 Feb 24;11:1117836. doi: 10.3389/fcell.2023.1117836 (PMC9999028; doi:10.3389/fcell.2023.1117836)
Supplement: Supplementary file 1 [file Table1.docx]

1. **Supplementary­­­ Data**

**Supplementary Methods**

**Amputation, tissue collection, and micro-computed tomography**

Adult 18-month old female CD1 mice were purchased from the Charles River Laboratory (Wilmington, MA) and were amputated and administered OAA using methods previously described in the Material and Methods. Digits were collected and fixed at specific timepoints from 18-month old mice and were scanned and quantified via Micro-CT as previously described in the Material and Methods.

**2 Supplementary Figures**

**
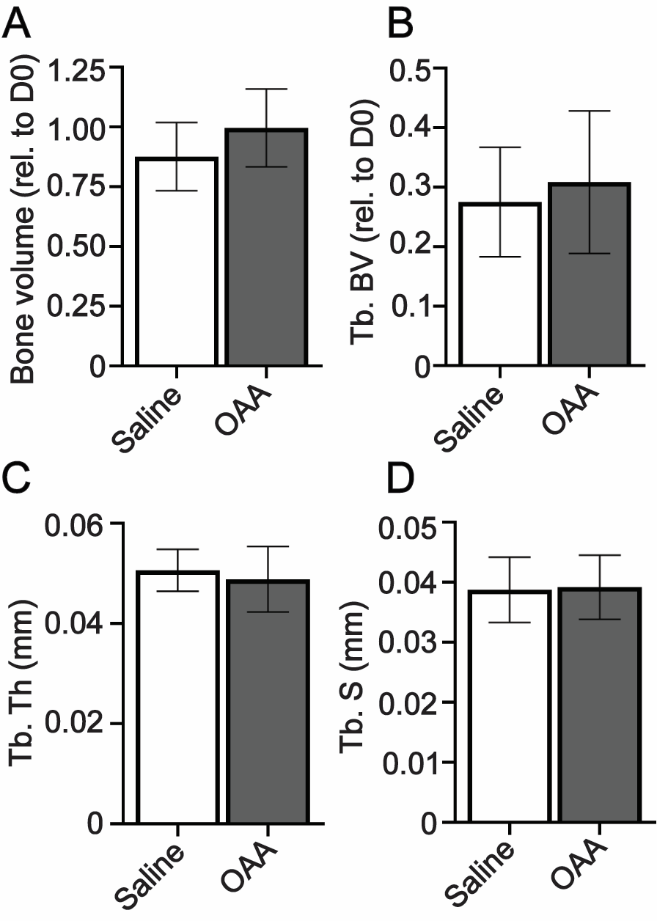
**

**Supplementary Figure 1. OAA administered to 18-month old mice from day 10 to 21.** Micro-CT quantification of (**A**) bone volume (relative to day 0), (**B**) trabecular bone volume (Tb. BV) (relative to D0), (**C**) trabecular thickness (Tb. Th), and (**D**) trabecular spacing (Tb. Sp). n=10-16 digits/group. Graphs represent average values±SD.


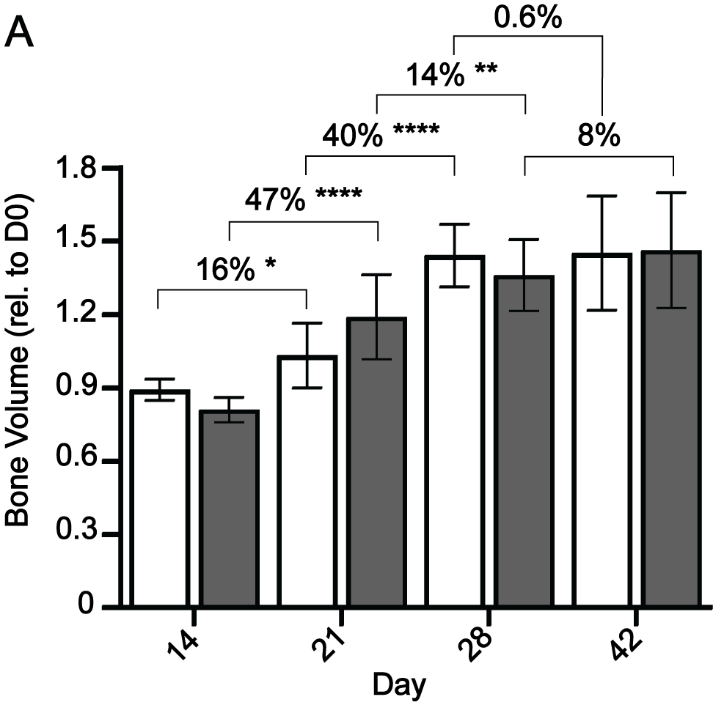


**Supplementary Figure 2. Precent increase of regenerated bone in 8-week old mice.** Micro-CT quantification of (**A**) bone volume (relative to day 0) in saline (Control) and OAA-treated mice. n=8-15 digits/group. Graphs represent average values±SD. *p<0.05, **p<0.01, ****p<0.0001.


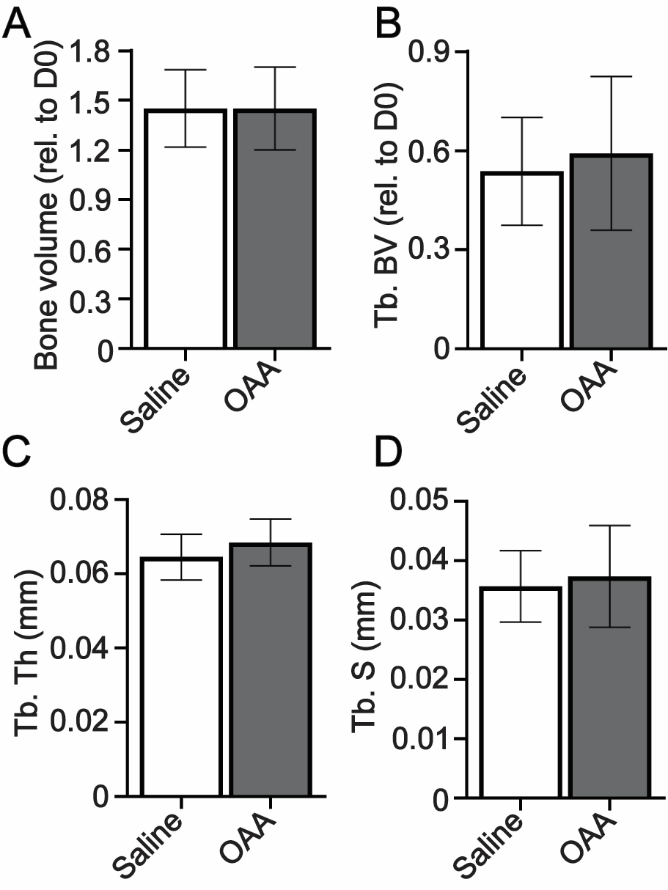


**Supplementary Figure 3. Evaluation of bone regeneration at day 42 in 8-week old mice after cessation of OAA dosing.** Micro-CT quantification of (**A**) bone volume (relative to day 0), (**B**) trabecular bone volume (Tb. BV) (relative to D0), (**C**) trabecular thickness (Tb. Th), and (**D**) trabecular spacing (Tb. Sp), at day 42 after OAA dosing from day 10 to 28. n=15 digits/group. Graphs represent average values±SD.


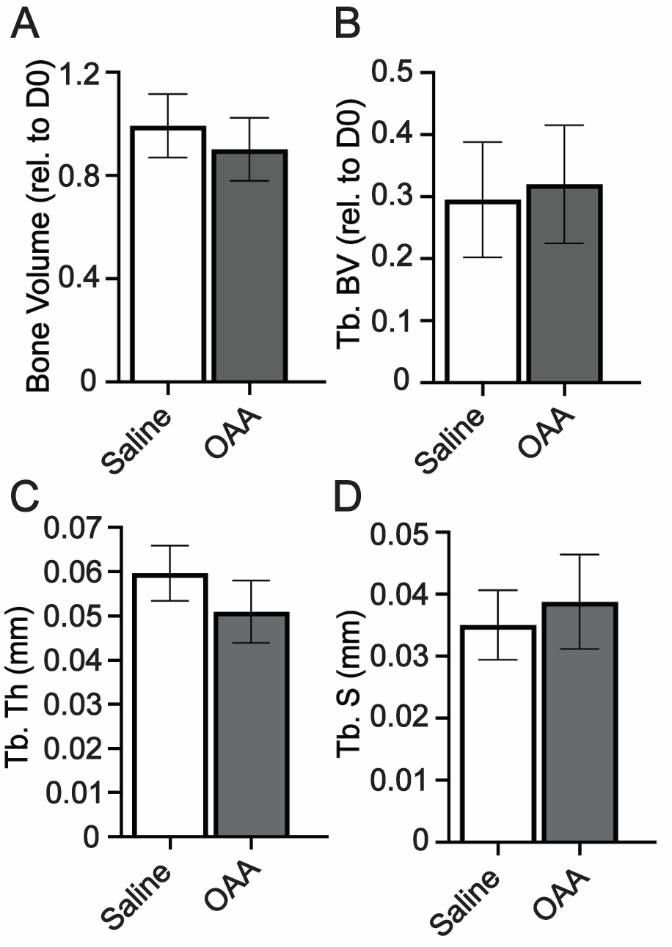


**Supplementary Figure 4. OAA administered to 18-month old mice from day 10 to 42.** Micro-CT quantification of (**A**) bone volume (relative to day 0), (**B**) trabecular bone volume (Tb. BV) (relative to D0), (**C**) trabecular thickness (Tb. Th), and (**D**) trabecular spacing (Tb. Sp). n=10-17 digits/group. Graphs represent average values±SD.
